# Characterization of Voltage-Gated Ca^2+^ Conductances in Layer 5 Neocortical Pyramidal Neurons from Rats

**DOI:** 10.1371/journal.pone.0004841

**Published:** 2009-04-01

**Authors:** Mara Almog, Alon Korngreen

**Affiliations:** 1 The Leslie and Susan Gonda Interdisciplinary Brain Research Center, Bar-Ilan University, Ramat Gan, Israel; 2 The Mina and Everard Goodman Faculty of Life Sciences, Bar-Ilan University, Ramat Gan, Israel; Instituto Cajal - CSIC, Spain

## Abstract

Neuronal voltage-gated Ca^2+^ channels are involved in electrical signalling and in converting these signals into cytoplasmic calcium changes. One important function of voltage-gated Ca^2+^ channels is generating regenerative dendritic Ca^2+^ spikes. However, the Ca^2+^ dependent mechanisms used to create these spikes are only partially understood. To start investigating this mechanism, we set out to kinetically and pharmacologically identify the sub-types of somatic voltage-gated Ca^2+^ channels in pyramidal neurons from layer 5 of rat somatosensory cortex, using the nucleated configuration of the patch-clamp technique. The activation kinetics of the total Ba^2+^ current revealed conductance activation only at medium and high voltages suggesting that T-type calcium channels were not present in the patches. Steady-state inactivation protocols in combination with pharmacology revealed the expression of R-type channels. Furthermore, pharmacological experiments identified 5 voltage-gated Ca^2+^ channel sub-types – L-, N-, R- and P/Q-type. Finally, the activation of the Ca^2+^ conductances was examined using physiologically derived voltage-clamp protocols including a calcium spike protocol and a mock back-propagating action potential (mBPAP) protocol. These experiments enable us to suggest the possible contribution of the five Ca^2+^ channel sub-types to Ca^2+^ current flow during activation under physiological conditions.

## Introduction

Pyramidal neurons of layer 5 in the neocortex are the primary output cells of the cortex [1]. They express a wide variety of voltage-gated ion channels, such as Na^+^, K^+^ and Ca^2+^ channels, whose differing distribution and density in the cell membrane determine the unique functioning of each cell [2], [3]. The channels that modulate many cellular processes are the voltage-gated Ca^2+^ channels. Voltage-gated Ca^2+^ channels are involved in electrical signalling and in converting electrical signals into cytoplasmic calcium changes [4]. Depolarization of the cell membrane causes the channels to conduct Ca^2+^ into the cytoplasm, raising the intracellular Ca^2+^ concentration. This increase, in turn, modulates cellular processes such as regulation of Ca^2+^-dependent channels, mediating neurotransmitter release, possibly influencing generation of action potentials [5], and stimulating intracellular signalling enzymes and gene expression [6], [7], [8], [9], [10], [11].

Several types of voltage-gated Ca^2+^ channels have been distinguished physiologically and pharmacologically. The channels can be distinguished physiologically both by the voltages which activate them and by whether they inactivate rapidly or not. For example, channels activating at relatively low voltages (low voltage-activated channels, LVA – T- and R- types (R-type activates at higher voltages than T-type, but lower than HVA channels)) inactivate rapidly. Channels requiring high voltages for activation (high voltage-activated, HVA) may display different inactivation rates [10], [12]. Pharmacological studies of mammalian brain neurons have revealed 4 types of HVA channels, L, N and P/Q [13].

Here we analyze the Ca^2+^ channels that can be found in single pyramidal cells to determine their possible contribution to the cell's physiological properties. Previous studies on cortical pyramidal cells have revealed 5 sub-types of Ca^2+^ current [14], [15], [16]. However, these experiments were carried out on dissociated neurons and, thus, possibly described Ca^2+^ channels in different types of cortical pyramidal neurons. The results obtained also depend on the developmental stage or age of the cells. During development of Layer 5 (L5) pyramidal neurons the density of Ca^2+^ channels increases in the apical dendrite, parallel to an increase of Ca^2+^ currents in the soma [15]. LVA current density decreases during the earliest postnatal development and HVA current density increases [17], [18]. Moreover, information about the activation of the various Ca^2+^ channels during action potentials and dendritic Ca^2+^ spikes is limited.

To unravel the role of voltage-gated Ca^2+^ channels in the back-propagating AP and the dendritic Ca^2+^ spike, we examined the properties of these channels in visually identified L5 neocortical pyramidal neurons. We developed recording conditions that allow us to record these channels in nucleated patches. Visually guided patch-clamp experiments in the slice preparation allowed us to target only L5 pyramidal neurons. Using these somatic nucleated patches, we were able to determine the sub-types, pharmacological properties, and kinetics of voltage-gated Ca^2+^ channels present in the soma membrane of these cells. We show that five Ca^2+^ channel sub-types (L-, N-, R- and P/Q-type) are expressed in the soma of these neurones. Finally, we applied voltage-clamp protocols that simulate the shape of the back-propagating AP and dendritic Ca^2+^ spike obtaining the activation profile of the various Ca^2+^ conductances during these physiological events. The contribution to overall current differed slightly for each channel sub-type (ranging from about 14–25%) and was independent of the stimuli used.

## Methods

### Slice preparation

Sagittal brain slices (300 µm thick) were prepared from the somatosensory cortex of 12–16 day old Wistar rats killed by rapid decapitation as described previously [19]. Slices were perfused throughout the experiment with an oxygenated artificial cerebrospinal fluid (ACSF) containing (mM): 125 NaCl, 25 NaHCO_3_, 2.5 KCl, 1.25 NaH_2_PO_4_, 1 MgCl_2_, 2 CaCl_2_, 25 glucose, 0.5 ascorbate (pH 7.4 with 5% CO_2_, 310 mosmol/kg). All experiments were carried out at room temperature (20–22°C). Pyramidal neurons from L5 in the somatosensory cortex were visually identified using infrared differential interference contrast (IR-DIC) videomicroscopy [19].

### Solutions and Drugs

The standard pipette solution contained (mM): 125 K-gluconate, 20 KCl, 10 HEPES, 4 MgATP, 10 Na-phosphocreatine, 0.5 EGTA, 0.3 GTP (pH 7.2 with KOH, 312 mosmol/kg). In experiments with Cs^+^ K-gluconate was replaced with the same amount of Cs-gluconate. In experiments with high concentrations of EGTA (10 mM) or BAPTA (1 mM), the equivalent amount of NaCl was removed from the pipette solution. The application solution contained (mM): 110 NaCl, 10 HEPES, 2.5 KCl, 1 MgCl_2_, 5 BaCl_2_, 25 Glucose, 5 4-AP, 20 TEA, 0.01 TTX. This application solution was used in all the nucleated patch experiments and was applied directly to the patch using a glass pipette. This allowed local perfusion of the patch with toxins and drugs. In experiments where the Ba^2+^ concentration was lower (3 mM) and/or was replaced with Ca^2+^ (2 mM), the equivalent amount of NaCl was added to the application solution to preserve osmotic pressure (TTX, tetrodotoxin, Alomone Labs, Jerusalem, Israel; TEA, tetraethylammonium, Sigma; 4-AP, 4-aminopyridine, Merck). The following toxins and blockers were used: nifedipine (Sigma) was diluted in 95% ethanol immediately before use and the application solution was protected from ambient light. Final ethanol concentration was fixed to 10 µM. ω-agatoxin IVA (ω-AgTx IVA) and ω-conotoxin GVIA (ω-CgTx GVIA) (Alomone Labs, Jerusalem, Israel), ω-conotoxin MVIIC (ω-CgTx MVIIC) and SNX-482 (Peptide Institute, Japan) were stored at −20°C as stock solutions in double distilled water. The application solutions with the different toxins and blockers were applied locally using perfusion tubing coated with Sigmacote (Sigma) to prevent binding of the toxins. In experiments with toxins, 0.1 mg/ml bovine serum albumin (BSA, Sigma) was added to the application solution to prevent non-specific binding. In current-clamp experiments the hyperpolarization-activated cation channels (I_h_ channels) were blocked by adding ZD7288 (NBT, Jerusalem, Israel) to the ACSF.

### Nucleated outside-out patches

Nucleated outside-out patches [20] were extracted from the soma of visually identified L5 pyramidal neurons. Suction (180–230 mbar) was applied when recording in the whole cell configuration and the pipette was slowly retracted. With gentle retraction it was possible to obtain large patches of membrane engulfing the nucleus of the neuron. Following the extraction of the patch the pressure was reduced to 30–40 mbar for the duration of the experiment. All measurements from nucleated patches were carried out with the Axopatch-200B amplifier (Axon Instruments, Foster City, CA). Nucleated patches were held at −60 mV. Linear leak and capacitive currents were subtracted on-line by a P/6 protocol taken at hyperpolarized voltages (−80 to −100 mV). Currents were filtered with 2–5 KHz and sampled at 10–50 KHz. The average series resistance was 13±0.3 MΩ (n = 187). The reference electrode was an Ag-AgCl pellet placed in the experimental chamber. Under these conditions the total voltage offset due to electrode and liquid junction potentials [21] was measured as −11 mV. Membrane potential was not corrected for this potential difference. When kinetic protocols were applied, the pipettes (4–7 MΩ) were coated with Sylgard (DOW Corning).

### Analysis

All off-line data analysis including curve fitting was carried out with IGOR (WaveMetrics, Lake Oswego, USA) on a PC computer. Experimental results were obtained from cells from two or more animals. All the results for a particular experiment were pooled and displayed as mean±S.E.M. Groups were compared using an unpaired t-test. Current traces were analyzed assuming a Hodgkin-Huxley model [22]. The activation and deactivation current traces were fitted to the general equation according to the Hodgkin and Huxley model [22]:
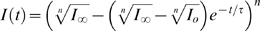
(1)where t is time, I_∞_ is the steady-state current, I_o_ is the current at t = 0, τ is the time constant of the exponential relaxation, and n is the number of gates in the model. Since I_o_ is close to zero at the holding potential prior to channel activation, the above equation simplifies to:
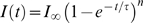
(2)


Correspondingly, I_∞_ is close to zero at the holding potential after the channels have finished deactivating. So in order to apply to tail currents equation 1 simplifies to
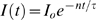
(3)


The current-voltage plots recorded from each patch were fitted to a Boltzmann equation:
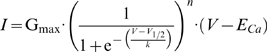
(4)where I/I_max_ is the current normalized to its maximal value, G_max_ is the maximal conductance, V is the membrane potential, V_1/2_ is the voltage at which the conductance is half-maximal (for a single gate, n = 1), k is the slope factor and E_Ca_ and E_Ba_ are the Ca^2+^ and Ba^2+^ reversal potential, respectively (when Ca^2+^ is replaced with Ba^2+^, E_Ca_ in the equation is replaced by E_Ba_). Using this equation produced better results than the accepted analysis protocol in which the conductance is first calculated from the current by dividing it with the driving force. Due to the positive reversal potential this traditional analysis method introduces large errors in the estimated value of the conductance as the voltage approaches the reversal potential. Fitting the I-V directly with equation 4 avoids this problem. To average the results obtained from several patches the I-V recorded in each patch was divided by the G_max_ obtained by fitting this individual I-V to equation 4 and the normalized I-Vs were averaged. Therefore, the I-Vs presented in the manuscript are plotted using an axis of I/G_max_.

## Results

### Recording voltage-gated Ca^2+^ conductances in nucleated patches

**Figure 1:**
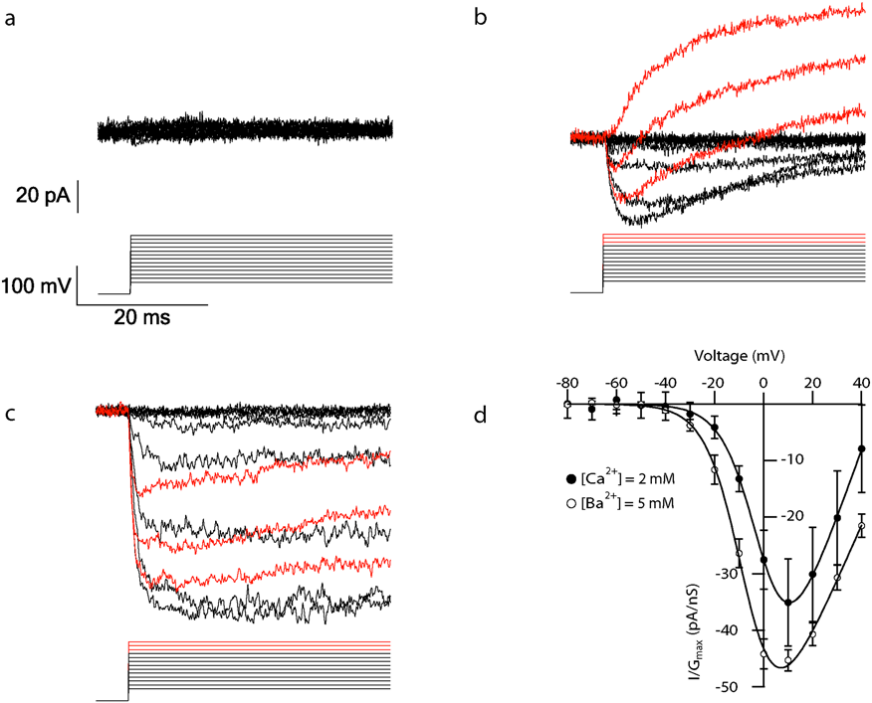
Ca^2+^ and Ba^2+^ currents recordings from nucleated patches. *a*, Currents recorded from a nucleated patch with a Cs-gluconate pipette solution and a Ca^2+^ (2 mM) application solution. A 500 ms pre-pulse to −110 mV was followed by a 100 ms pulse to voltages between −80 and +40 mV at 10 mV increments. The −110 mV pre-pulse was truncated to facilitate the display of the current. Records were sampled at 20 KHz and filtered at 5 KHz. Leak was subtracted on-line. The voltage protocol is shown below the current traces. *b*, Inward and outward currents from a nucleated patch using a K-gluconate pipette solution and a Ca^2+^ (2 mM) application solution (see methods). The overlapping traces are marked in red in order to highlight them. The voltage protocol and scale bar as in *a*. The voltage protocol is shown below the current traces. *c*, Inward currents from a nucleated patch using a K-gluconate pipette solution and a Ba^2+^ (5 mM) application solution (see methods). The overlapping traces are marked in red in order to highlight them. The voltage protocol and scale bar as in *a*. The voltage protocol is shown below the current traces. *d*, Mean activation curves of the Ca^2+^ current in *b* (•, n = 6) and the Ba^2+^ current in *c* (○, n = 5). The currents were normalized to the maximal conductance at a given series of voltages. The smooth lines are the fit to a Boltzmann function with one gate with a V_1/2_ of 0±1 mV, k = 7.2±0.2 mV, E_Ca_ = 47±1 mV for the Ca^2+^ currents (•) and a V_1/2_ of −7±1 mV, k = 7.3±0.2 mV, E_Ba_ = 62±1 mV for the Ba^2+^ currents (○). Error bars are S.E.M.

Pipette solutions substituting Cs^+^ ions for K^+^ ions are traditionally used to measure voltage-gated Ca^2+^ currents in the patch-clamp technique [11], [23]. Therefore, we first extracted nucleated patches from the cell using a Cs^+^ pipette solution. The patch was then positioned in front of an application solution containing 2 mM Ca^2+^ ions, 10 µM tetrodotoxin (TTX) to block voltage-gated sodium currents, 20 mM tetraethylammonium (TEA) and 5 mM 4-aminopyridine (4-AP) to block K^+^ currents. No voltage-gated Ca^2+^ currents were observed using this pipette solution (Fig. 1a, n = 5). Next, we attempted the same experiment using a pipette solution containing K^+^ ions. This procedure revealed voltage-gated Ca^2+^ currents (Fig. 1b). Although K^+^ blockers were added to the application solution, the recording was contaminated by voltage-gated K^+^ currents (Fig. 1b). Our initial approach was to block the Ca^2+^ current with 50 µM Cd^2+^ in order to obtain clean Ca^2+^ traces by subtraction of the remaining K^+^ currents from the total current (Fig. 1b). This approach was not successful due to differences between the K^+^ currents recorded before and after the application of Cd^2+^. This may be due to the presence of Ca^2+^ dependent K^+^ conductances in the patch. Regardless of the cause, this prohibited simple subtraction of the K^+^ current.

To reduce the contamination by K^+^ currents and increase the amplitude of the inward current we replaced Ca^2+^ ions (2 mM) in the application solution with Ba^2+^ ions (5 mM) (Fig. 1c). As in previous reports, the Ba^2+^ currents obtained with 5 mM Ba^2+^ exhibited similar voltage-dependence to those obtained with 2 mM Ca^2+^ [16]. Figure 1d shows the mean normalized activation curves of the Ca^2+^ currents (filled circles, n = 6) and Ba^2+^ currents (empty circles, n = 5). The curve was fitted to a Boltzmann fit function assuming one activation gate (smooth lines) and gave a V_1/2_ of 0±1 mV, k = 7.2±0.2 mV, E_Ca_ = 47±1 mV for the Ca^2+^ currents and a V_1/2_ of −7±1 mV, k = 7.3±0.2 mV, E_Ba_ = 62±1 mV for the Ba^2+^ currents. The V_1/2_ and the k values of the Ca^2+^ and the Ba^2+^ currents are similar but the Ba^2+^ reversal potential was about 20 mV higher. The difference in the reversal potential can probably be explained by K^+^ currents contamination of the Ca^2+^ current. Because of the similarity of the activation curves and the fact that Ba^2+^ increased the current amplitude, Ba^2+^ application was used in all experiments.

### Runup and rundown of Ba^2+^ currents

**Figure 2:**
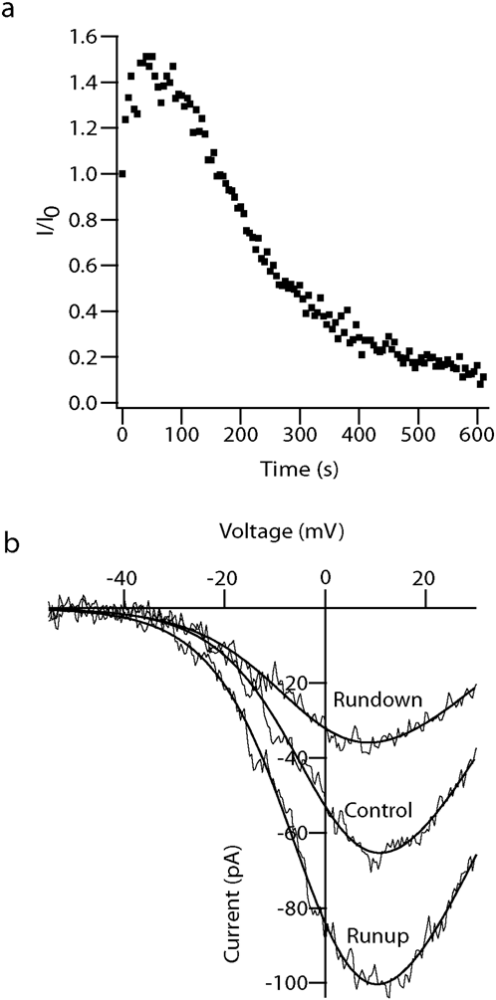
Runup and rundown of Ba^2+^ currents. *a*, The normalized current of one nucleated patch as a function of time. t = 0 indicates the rupture of the membrane separating the pipette solution from the cell and its positioning in front of the Ba^2+^ application solution. The pipette solution contained 0.5 mM EGTA. The currents were recorded using a ramp protocol from −100 mV to +80 mV for 50 ms with a time interval between the protocols of 5 seconds. Records were sampled at 10 KHz and filtered at 2 KHz. Leak was subtracted on-line. *b*, Activation curves of Ba^2+^ currents obtained at t = 0 (control), t = 47 s (runup) and t = 273 s (rundown) in the experiment shown in *a*. The smooth lines are the fit to a Boltzmann function with one gate to the current obtained at time 0 (control), after 47 seconds (runup) and after 270 seconds (rundown). This fit gave a mean G_max_ of 2.7±0.3 nS, V_1/2_ of 3±1 mV, k = 7.5±0.6 mV, E_Ba_ = 43±2 mV for the control current (n = 15), a mean G_max_ of 3.2±0.3 nS, V_1/2_ of −2±1 mV, k = 7.7±0.3 mV, E_Ba_  = 46±2 mV for the runup current (n = 17) and a mean G_max_ of 2.3±0.2 nS, V_1/2_ of −2±3 mV, k = 7.6±0.5 mV, E_Ba_ = 44±2 mV for the rundown current (n = 16).

Ca^2+^ currents have a tendency to decline with time starting with patch excision (“rundown”) [23]. This decline was also observed in the Ba^2+^ currents (Fig. 2a). However, a current increase occurred during the first 1–2 minutes of the recordings (Fig. 2a). This enhancement in the Ba^2+^ current may result from facilitation of Ca^2+^ channels. Previous studies have reported both rapid facilitation (after only few milliseconds,[24]) and slow facilitation (ranging from 5–10 minutes [25] to about 1 hour [26]). As our findings did not match these observations, it is more likely that this current enhancement was not caused by facilitation but by runup of these channels, as previously reported [27], [28].

Figure 2a displays the peak current of a nucleated patch obtained using a 50 ms ramp protocol from −100 mV to +80 mV that was repeated every 5 seconds. The current increased, followed by a decline beginning after 50 seconds and terminating with zero Ba^2+^ current after 600 seconds. The time for reaching zero current was defined in each experiment as the time in which the current amplitude reached 10% of its initial value. Below 10% of the initial amplitude it was not possible to differentiate between signal to noise (these rundown kinetics were observed in every patch with a standard deviation of 200 seconds, n = 21). This rundown left a time window of about 2–5 minutes in which quantitative recordings could be performed.

In an attempt to slow the rundown we first increased the time interval between the pulses from 5 to 10 and 20 seconds, since stimulation of less than 1 Hz has been reported to reduce rundown [23]. Next, the EGTA concentration in the pipette solution was increased from 0.5 mM to 10 mM. Then 1 mM BAPTA was added to the 10 mM EGTA pipette solution [23], [29]. None of these modifications changed the rate of runup or rundown of the Ba^2+^ currents (data not shown). We then examined the kinetics of the Ba^2+^ currents during runup and rundown (Fig. 2b) using voltage-ramps followed by curve fitting to a Boltzmann function assuming one activation gate. There were no significant differences in the kinetics of runup and rundown. Thus, the rundown may result from decrease in the number of channels available for activation (especially in an isolated membrane patch) rather than a change in the conductance of a single channel or in the open probability of the channels.

### Kinetics

**Figure 3:**
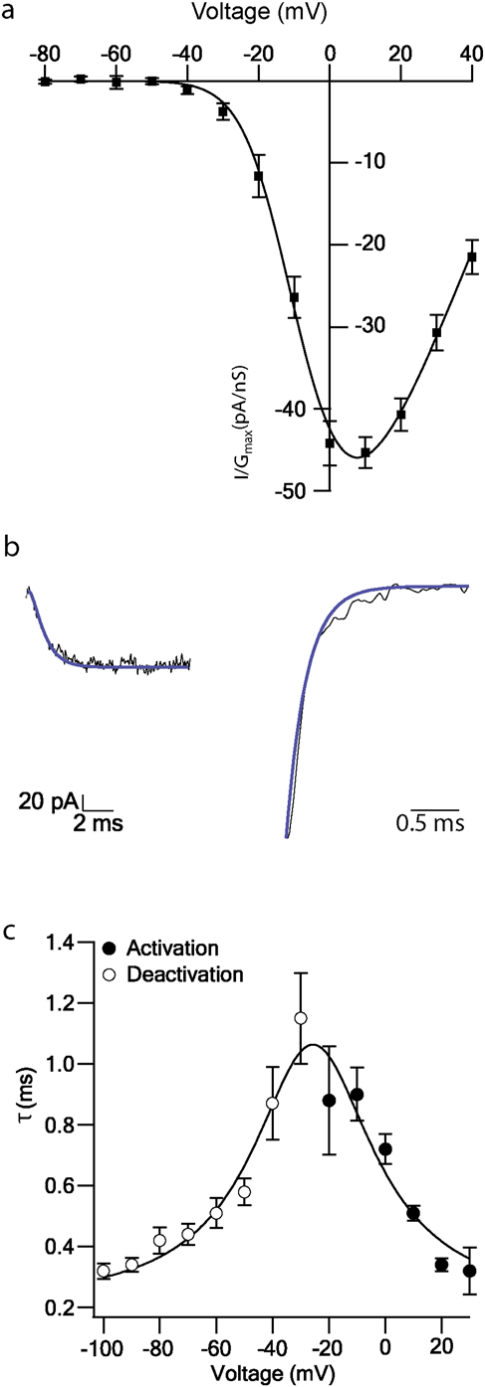
Activation and deactivation of Ba^2+^ currents in nucleated patches. *a*, Activation curve of the Ba^2+^ current. The mean currents were normalized to the maximal conductance for a given series of voltages (n = 5). The smooth line is the fit to a Boltzmann function with two gates with a V_1/2_ of −14.2±0.6 mV, k = 9.8±0.6 mV, E_Ba_ = 59±2 mV. Error bars are S.E.M. *b*, Activation (left) and deactivation (right) fitting of a second order Hodgkin-Huxley model (thick line). *c*, Activation (•, n = 10) and deactivation (○, n = 8) time constants determined from traces like those in *b*. The smooth line is the curve fit to the equation: C_1_+C_2_/((V-C_3_)^2^+C_4_), where C_1_ is the time constant at 0 voltage, C_2_ is the height of the equation peak, C_3_ is the voltage at the center of the peak and C_4_ is the standard deviation. The fit gave a C_1_ = 0.20±0.08 ms, C_2_ = 0.6±0.2 ms*mV^2^, C_3_ = −25.7±1.7 mV and C_4_ = 0.7±0.2 mV^2^. Errors bars are S.E.M.

The activation kinetics of the Ba^2+^ current were examined with the voltage-clamp protocols shown in figures 1a–c. Figure 3a shows the mean normalized activation curves of the Ba^2+^ current (n = 5). In many studies the voltage-gated Ca^2+^ channel kinetics were described by two activation gates and one inactivation gate (the m^2^ h model [22]). Correspondingly, all the kinetic analysis performed in this study conformed to this model. The use of a single gate model in the previous sections was performed to allow visual comparison between the traces and the fit results. A Boltzmann fit function of two gates was fitted (smooth line), giving a V_1/2_ of −14.2±0.6 mV, k = 9.8±0.6 mV and E_Ba_ = 59±2 mV. Deactivation was measured by a pre-pulse of −100 mV followed by a depolarization step of +10 mV for 2 ms and 30 ms 10 mV hyperpolarization steps from −30 mV to −100 mV. A second order Hodgkin-Huxley model was fitted to the activation and the deactivation traces, i.e. to the decay phase of the current and the rising phase of the current, respectively (Fig. 3b). These fits gave a time constant (τ) for each voltage at which the membrane was held. Figure 3c shows the mean time constants for the activation (n = 10) and the deactivation (n = 8). The time constants extracted from this analysis ranged from 0.32±0.08 ms at +30 mV (Fig. 3c, n = 10) to 0.32±0.03 ms at −100 mV (Fig. 3c, n = 8) and displayed a bell-shaped dependence on voltage.

**Figure 4:**
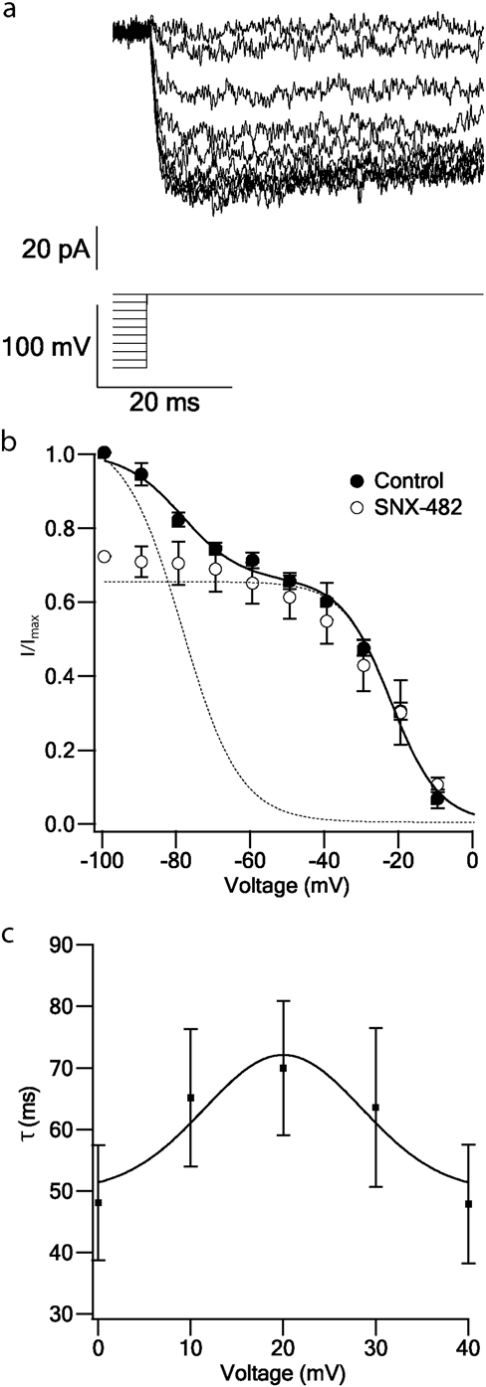
Inactivation kinetics of Ba^2+^ currents in nucleated patches. *a*, Inactivation of inward currents recorded from a nucleated patch using Ba^2+^ application solution. Inward current was generated by a 150 ms pulse to voltages between −90 and 0 mV with 10 mV increments. The patch was subjected to a 50 ms depolarising step to 0 mV (the voltage protocol is shown below the traces). The voltage was stepped to −80 mV for 50 ms after every sweep to allow Ca^2+^ channels to recover from inactivation (not shown). Records were sampled at 50 KHz and filtered at 2 KHz. Leak was subtracted on-line. *b*, Mean inactivation curve of the control current (•, n = 7) and the current remained after application of the blocker SNX-482 (○, n = 4). The peak current was normalized to the maximal current obtained from a series of pulses in the control conditions. The smooth line is the line calculated using a combination of two Boltzmann functions with one gate. The fit gave a first V_1/2_ of −79±3 mV and a second V_1/2_ of −23±2 mV, a k_1_ = −8±3 mV and a k_2_ = −7±1 mV. The dash lines are the separated Boltzman functions fitted to the control current. Errors bars are S.E.M. c, Mean inactivation time constant calculated from the rising phase of the activation currents which were recorded using Ca^2+^ application solution (n≥8). The smooth line was calculated using a fit of the equation C_1_+C_2_*exp(-((V-C_3_)/C_4_)^2^), where C_1_ is the time constant at 0 voltage, C_2_ is the height of the Gaussian peak, C_3_ is the voltage at the center of the peak and C_4_ is the standard deviation. This fit gave a C_1_ = 50 ms, C_2_ = 22.10 ms, C_3_ = 20 mV and C_4_ = 12 mV. Errors bars are S.E.M.

The steady-state inactivation of the total current was measured using a 150 ms conditioning pulse to voltages between −90 and 0 mV with 10 mV increments. The patch was then subjected to a 50 ms depolarising step to 0 mV to record the remaining current (Fig. 4a). Figure 4b shows the steady-state inactivation curve. The current amplitude was normalized to the maximal current obtained in each patch, and the control curve is a mean of 7 patches (Fig. 4b). As we observed two inactivation components a sum of two Boltzman functions was fitted (smooth line). The fit gave two V_1/2_ the first one being −79±3 mV and the second one −23±2 mV, a k_1_ = −8±3 mV and a k_2_ = −7±1 mV. It has been shown that T-type and R-type channels inactivate at low voltages [30], [31], but they both differ in their activation threshold [32]; T-type activates at −60 mV [33], [34] whereas R-type activates at −30 mV [35]. In our experiments, no T-type currents were observed neither in the activation protocol nor in the ramp protocol at voltages around −60 mV. This suggested that the low voltage component of the inactivation curve may be due to the R-type conductance. To test this possibility we measured the steady-state inactivation of the current when the R-type blocker, SNX-482 (30 nM) was added to the application solution. At this blocker concentration the first component of the inactivation was almost completely eliminated and the remaining current corresponded to HVA channels (Fig. 4b). Thus, the first V_1/2_ may correspond to R-type channels [31] and the second V_1/2_ probably corresponds to the HVA channels.

The inactivation time constant (Fig. 4c) was measured from the rising phase of the Ca^2+^ current obtained by an activation protocol (Fig. 1b). The inactivation shape with Ca^2+^ application solution differed from that obtained with Ba^2+^ application solution. The inactivation phase of the Ca^2+^ current (Fig. 1b) was steeper than the activation of the Ba^2+^ current (Fig. 1c). This difference can arise from the absence of calcium-dependent inactivation in the Ba^2+^ application, the remaining inactivation being voltage-gated only.

### Pharmacology

**Figure 5:**
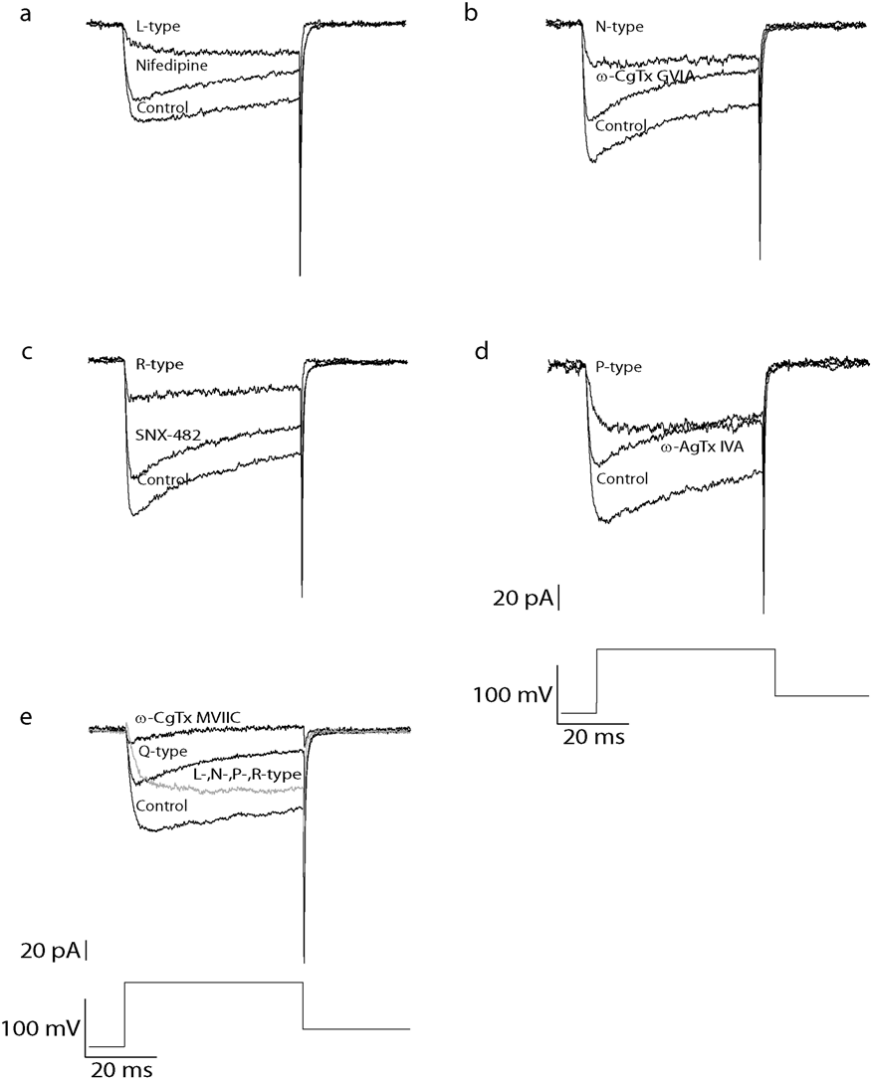
Pharmacological separation of the 5 Ba^2+^ current sub-types with Ca^2+^ channel blockers. *a*, Currents evoked by a 50 ms step depolarization to 0 mV from a holding potential of −110 mV before (control) and after application of 10 µM nifedipine. The nifedipine-sensitive current (L-type) was obtained by subtraction. *b*,*c*,*d* and *e*, Same stimulation protocol as in *a*. *b*, 1 µM ω-CgTx GVIA (N-type blocker) was added to the application solution. *c*, 30 nM SNX-482 (R-type blocker) was added to the application solution. *d*, 200 nM ω-AgTx IVA (P-type blocker) was added to the application solution. *e*, the control current was recorded with an application solution containing blockers for L-, N-, R- and P-type. In order to eliminate the remaining current 1 µM ω-CgTx MVIIC (Q-type blocker) was added to the application solution.

The results obtained using the inactivation protocol suggested that the R-type voltage-gated Ca^2+^ channel sub-type was expressed in the somatic membrane of L5 pyramidal neurons (Fig. 4b). Next we attempted to pharmacologically dissect out the relative contribution of the different voltage-gated Ca^2+^ channels. Ba^2+^ currents were elicited by a 50 ms step depolarization to 0 mV from a holding potential of −110 mV (Fig. 5). L-, N-, R- and P-type currents were blocked by 10 µM nifedipine (Fig. 5a), 1 µM ω-CgTx GVIA (Fig. 5b), 30 nM SNX-482 (Fig. 5c) and 200 nM ω-AgTx IVA (Fig. 5d), respectively [36], [37], [38], [39]. Q-type current was blocked by 1 µM ω-CgTx MVIIC [40]. This blocker is not only Q-type selective but may also block N-, and P-type currents [41]. To isolate the Q-type current the blockers for L-, N-, R- and P-type channels were added to the application solution before blocking the Q-type channel (Fig. 5e). After blocking each sub-type channel we exposed the patch to an application solution containing 50 µM Cd^2+^. This blocked the remaining current in every patch, regardless to the blocker used (data not shown). In this study we have not observed currents that were, given the experimental signal to noise ratio, resistant to Cd^2+^.

Since T-type Ca^2+^ channels have a relatively low activation threshold, T-type currents may be isolated kinetically. No T-type currents were observed in our kinetic experiments. To confirm that T-type channels are not expressed in L5 pyramidal neurons, current-clamp experiments were carried out in the whole-cell configuration. This was done to test whether the rebound firing in the cells could be caused by T-type channels that activate at low voltages [42], [43], although this is mostly caused by the hyperpolarization-activated cation channels (I_h_ channels) [44]. The membrane potential was measured with and without Cd^2+^. ZD7288 was then added to the ACSF and the membrane potential was measured again. The rebound firing vanished only after addition of ZD7288 (data not shown). This suggests that T-type channels are not expressed by these neurons, at least not in the soma membrane nor the membrane near it.

**Figure 6:**
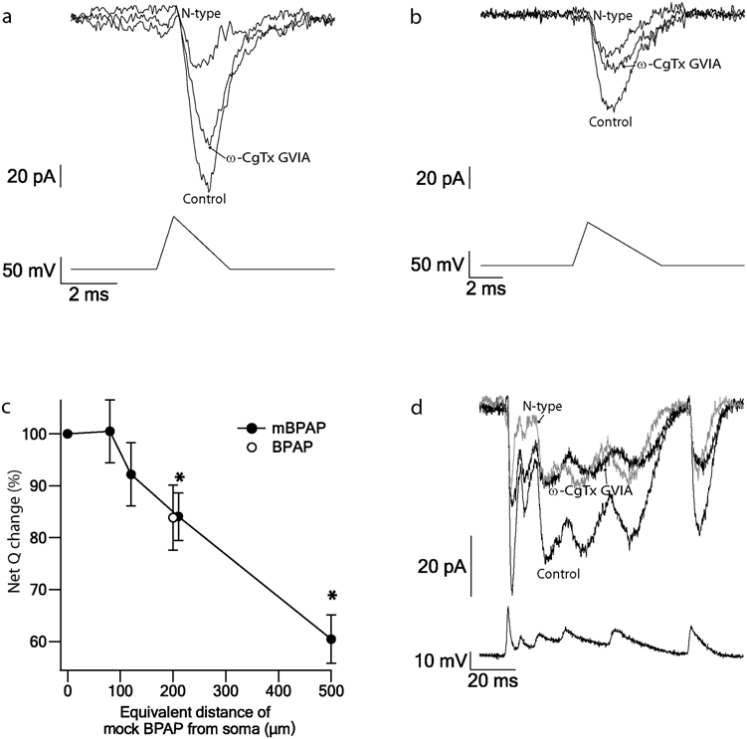
Ba^2+^ currents recorded using different physiological pulses. *a*, A mBPAP generated using parameters of somatic AP was used as a voltage-clamp command in the nucleated patch (bottom, the rise phase of the action potential was simulated by a 0.6 ms ramp from a holding potential of −60 mV to +40 mV and the repolarization phase of an action potential is simulated by a 2 ms ramp from +40 mV to the holding voltage potentia). This evoked a Ba^2+^ current. Shown are the current evoked by the mBPAP before (control) and after 1 µM ω-CgTx GVIA (N-type blocker). The ω-CgTx GVIA-sensitive current (N-type) was obtained by subtraction. *b*, A mBPAP generated to simulate a back-propagating AP at the dendrite about 170 µm from the soma was used as a voltage-clamp command in the same nucleated patch as in a (bottom similar ramps to that described in A were used to simulate a back-propagating AP. In order to simulate the amplitude decay and the half with increase of a back-propagating AP, the maximal ramp amplitude was reduced by 6 mV in each step and the time of the rising and decline ramps was increased by 0.1 ms and 0.8 ms in each step, respectively). As in a, this evoked a Ba^2+^ current shown here before (control) and after 1 µM ω-CgTx GVIA (N-type blocker). The ω-CgTx GVIA-sensitive current (N-type) was obtained by subtraction. *c*, The net average charge (Q) displayed as a percentage of the first mBPAP (control) (•, n = 14). A back-propagating action potential measured at 200 µm in these cells was used as a voltage-clamp command applied to the patched and is displayed as a percentage of the action potential generated at the soma (○, n = 4). The data is plotted as a function of the equivalent distance of mBPAP from the soma in µm. Error bars are S.E.M. The asterisk indicates a significant difference (p<0.005, one-tail t-test) between the mAP at the soma from the different mBPAPs along the dendrite. *d*, A Ca^2+^ spike as recorded at the distal dendrite (550 µm from the soma) of a L5 pyramidal neuron was used as a voltage-clamp command in the same nucleated patch as in a, (bottom). The Ca^2+^ spike was 140 ms long. This evoked a Ba^2+^ current, shown here before (control) and after 1 µM ω-CgTx GVIA (N-type blocker). The ω-CgTx GVIA-sensitive current (N-type, grey) was obtained by subtraction.

Having established the recording conditions and examined the pharmacology of the Ba^2+^ currents in nucleated patches, we next examined the activation of the channels under conditions of more physiologically realistic voltage-clamp protocols. In the apical dendrite L5 pyramidal neurons voltage-gated Ca^2+^ channels are activated by back-propagating action potentials [45] and dendritic Ca^2+^ spikes [5], [45]. First, we designed voltage-clamp protocols that simulated the shape of back-propagating AP. It is well known that there is a high variability in the shape and amplitude of an action potential that propagates from the soma along the apical dendrite [3], [46], [47]. In order to avoid these variances we extracted the parameters of a back-propagating action potential (amplitude, half-width and time to peak) from previous studies [46], [48] and generated a protocol that simulates back-propagating action potential. The protocol was constructed of a rising ramp to +40 mV from a resting potential of −60 mV and a slower decaying ramp. Both ramps changed the amplitude and delay values as the stimuli simulate the action potential that back-propagate along the apical dendrite. The shape of the dendritic Ca^2+^ spike is even more variable than the shape of the back-propagating AP [49]. Thus, instead of generating a noise free mock protocol we used the waveform of a dendritic Ca^2+^ spike recorded by us at 550 µm along the apical dendrite as a voltage-clamp command. Figure 6 shows Ba^2+^ currents measured applying a mock back-propagating action potential protocol (mBPAP, figs. 6a–b) and a Ca^2+^ spike protocol (Fig. 6d). The Ca^2+^ spike recorded from a L5 pyramidal neuron generated a high frequency burst of four APs at the soma. For comparison of the Ca^2+^ influx, the single AP was triggered immediately after the Ca^2+^ spike. A series of mBPAPs were used as voltage-clamp commands, the first having the properties of a somatic action potential in L5 pyramidal neurons while the following spikes had properties similar to back-propagating APs at different distances from soma [46], [48]. This protocol was applied before and after application of the different blockers for the various Ca^2+^ channel sub-types. Figure 6a shows the N-type current together with the control current when a mAP similar to the AP recorded at the soma was used as a voltage-clamp command. Figure 6b shows the N-type current of the same patch as in figure 6a when a mBPAP with properties similar to the back-propagating AP recorded 210 µm from soma was used as a voltage-clamp command. There was a significant decrease in the Ba^2+^ current as the mBPAP simulated back-propagating APs recorded further from the soma (Fig. 6c). To validate the use of the mBPAP protocols we recorded one back-propagating AP from 200 µm along the apical dendrite of a L5 pyramidal neuron and used it as a voltage-clamp command in addition to the mBPAPs. The currents measured with this protocol displayed similar current shape and amplitude to the one obtained with the mBPAP protocol simulating the action potential at 210 µm (traces not shown, average response id give in Fig. 6c). A Ca^2+^ spike measured from the apical dendrite of cortical L5 pyramidal neurons was used as a voltage-clamp command to the same patch as in figures 6a–b. Figure 6d shows the N-type current measured following this protocol.

**Figure 7:**
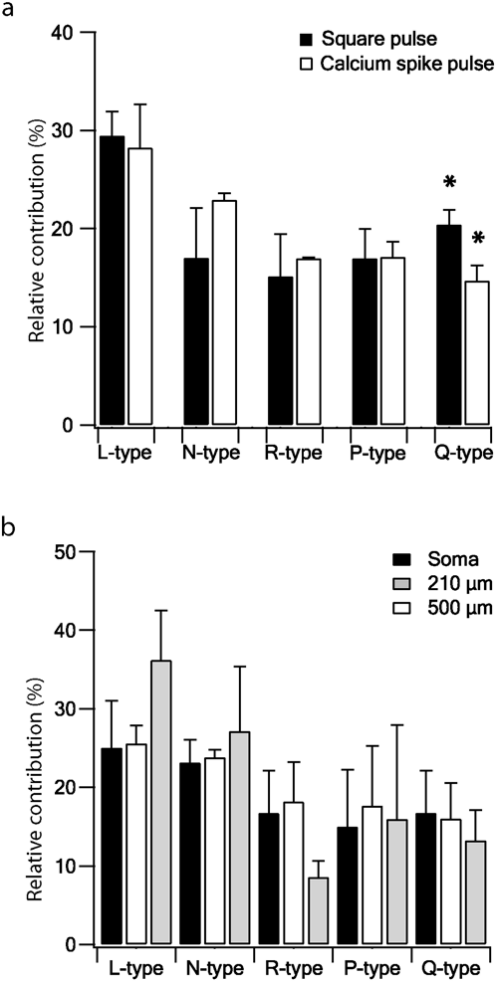
The contribution of Ba^2+^ current sub-types for different pulses to nucleated patch currents in neocortical L5 pyramidal neurons. *a*, The net average charge (Q) for each channel sub-type evoked by a square pulse (black bars) and a Ca^2+^ spike pulse (white bars) is displayed in the histogram as a percentage of the control Ba^2+^ current. The sum of the contribution of all the channel sub-types is higher than 100%, possibly due to the rundown observed or because the blockers for each channel sub-type blocked other sub-types as well. Thus, the contribution of each channel sub-type to the total current in the different protocols was plotted as the percentage of the sum of the 5 channel sub-type currents which was normalised to 100%. The square pulse gave a channel distribution of 29.5±2.4%, n = 7 for L-type; 17±5%, n = 3 for N-type; 16.2±4.3%, n = 4 for R-type; 17±3%, n = 3 for P-type; 20.4±1.5%, n = 4 for Q-type. The Ca^2+^ spike pulse gave a channel distribution of 28.2±4.4%, n = 6 for L-type; 22.9±0.7%, n = 2 for N-type; 17±0.1%, n = 3 for R-type; 17.1±1.6%, n = 2 for P-type; 14.7±1.6%, n = 2 for Q-type. Error bars are S.E.M. The asterisk indicates a significant difference (p<0.05, one-tail t-test) between the two different pulses. *b*, The contribution (percent) of each channel sub-type to the current evoked by a mBPAP protocol (calculated as in a). The percent contribution are displayed for 3 different mBPAP, simulating a somatic action potential (black bars), a back-propagating AP at 210 µm (white bars) and a back-propagating AP at 500 µm from the soma (grey bars). Error bars are S.E.M.

The contribution of each channel sub-type was calculated for the different protocols used (Fig. 7). Further, the net charge (Q) of the current recorded during the control or during a pharmacological application was calculated for each protocol. The percent contribution of each channel sub-type is derived as the mean of several patches. Figure 7a shows the relative contribution of each channel sub-type for the square step voltage (black bars) and for the Ca^2+^ spike protocol (white bars). Only the Q-type channel showed a significantly higher relative contribution in the square step than in the Ca^2+^ spike protocol (p<0.05, one-tail t-test). There was no significant difference in the relative contribution of each channel sub-type under the mBPAP protocol (Fig. 7b); that is, the relative contribution of each sub-type was similar when the different action potentials as seen in the soma and along the apical dendrite were used as voltage-clamp commands.

## Discussion

In this study we recorded voltage-gated Ca^2+^ conductances in the soma of visually identified L5 pyramidal neurons in acute brain slices from two-week old rats. We first developed the appropriate protocol for characterizing the properties of these channels and then examined the activation kinetics of the general Ba^2+^ current. No T-type channels were found but steady-state inactivation protocols in combination with pharmacology revealed the expression of R-type channels. Using pharmacological dissection and three different stimulus protocols – a square step depolarization, a calcium spike protocol and a mBPAP protocol – we identified 5 voltage-gated Ca^2+^ channel sub-types expressed in the soma membrane and determined their contributions to the overall current in the soma membrane.

### Kinetic properties of the voltage-gated Ca^2+^ channels

It is interesting to discuss first the modifications we had to perform in order to enable stable recordings of calcium currents from nucleated patches. The traditional pipette solution for recording Ca^2+^ currents is based on caesium. It was indeed a surprise to us that this solution did not work (Fig. 1). Only by replacing the caesium in the pipette solution with potassium were we able to observe calcium currents. Differences between the amplitude of the estimated conductance density of voltage-gated K^+^ conductances have been observed between studies performed using nucleated patches [50] and whole-cell [51]. However, to the best of our knowledge, there is no other study reporting problems with the caesium solution or suggested a mechanism for such an effect.

The activation of voltage-gated Ca^2+^ channels was similar using either Ca^2+^ or Ba^2+^ solutions (Fig. 1d). The Ba^2+^ solution eliminated K^+^ currents and produced sufficiently large and clean currents that repetition of the same protocol several times for averaging was unnecessary (Fig. 1c). Although, the activation properties of the current were not different during the rundown (Fig. 2b), the rundown interfered with measuring the various kinetics protocols applied allowing a time window of 5 minutes of stable recording before the signal to noise ratio became too big. The activation and deactivation properties of the Ba^2+^ current were very similar to those measured in other cells using different modes of the patch-clamp technique [14], [23], [52], [53]. The voltage required to activate half of the channel population (V_1/2_) was −14.2±0.6 mV and the slope was 9.8±0.6 mV, with a reversal potential of 59±2 mV (Fig. 3a).

The steady-state inactivation Ba^2+^ current was measured using the Ba^2+^ solution, which produced a slower inactivation than the Ca^2+^ solution (Figs. 1b–c), due to elimination of calcium-dependent inactivation. This phenomenon made it possible to measure voltage-dependent inactivation in isolation (Fig. 4a). The steady-state inactivation Ba^2+^ current revealed two components, the first with a V_1/2_ of −79±3 mV and the second a V_1/2_ of −23±2 mV (Fig. 4b). The experiments using the R-type blocker, SNX-482 (Fig. 4b), and the previously reported V_1/2_ of −82 mV [31] indicate that R-type channel is expressed in these neurons. Most of the previous studies on voltage-gated Ca^2+^ channels in cortical L5 pyramidal neurons have been performed in dissociated neurons using the whole cell configuration [14], [15], [16], [54] reporting similar kinetics and pharmacological properties of the voltage-gated Ca^2+^ channels to those obtained here.

According to the steady-state inactivation curve, R-type channels are ∼90% inactivated around the resting membrane potential. This still allows them to generate current following depolarization of the neuron. The measurements presented in figures 6 and 7 suggest that this current forms approximately 15% of the total Ca^2+^ current recorded in the nucleated patches. This may indicate that following substantial hyperpolarization of the neurons a larger current will flow via R-type channels. This may have implications on the generation and duration of dendritic Ca^2+^ spikes. It is tempting to speculate that this predicted variability in the Ca^2+^ current may be one of the factors contributing to the observed variability of dendritic Ca^2+^ spike shapes in the apical dendrite of L5 pyramidal neurons [49].

### Pharmacological properties of the voltage-gated Ca^2+^ channels

The presence of the R-type voltage-gated Ca^2+^ channel in the kinetic experiments led us to determine pharmacologically which of the different Ca^2+^ channel sub-types were present. Using the specific blockers for each channel sub-type (see methods) and a square depolarizing voltage step protocol (Fig. 5), we could show that these neurons express all the 4 HVA Ca^2+^ channels and the R-type channel but lack the T-type channel. These results agree with previous reports that cortical pyramidal neurons displayed an increase of HVA current density after the first period of postnatal development [17], [18].

We then tested the contribution of each channel sub-type to the overall Ba^2+^ current under varying stimulation protocols. The first protocol applied was a mBPAP (Figs. 6a–b); that is, a series of mBPAPs were used as voltage-clamp commands, the first having the shape of the action potential measured at the soma and the following potentials simulating an action potential back-propagating along the apical dendrite [55]. The measured Ba^2+^ currents decreased as the stimulating mBPAP “occurred” further along the dendrite (Fig. 6c). This phenomenon was observed for all the Ba^2+^ current sub-types and may be simply explained by the amplitude decrease of the mBPAP. This decrease was also observed when a back-propagating action potential measured from a L5 pyramidal neuron was used as a voltage-clamp command (Fig. 6c). The same contributions were obtained from the various mBPAP stimuli that simulated a back-propagating spike at different distances from the soma. While the normalized contribution of the L-type channels was close to 30%, that of the other 4 channel sub-types ranged between 14–25%. The same contributions were found under all three stimulation protocols (Fig. 7).

These results are consistent with Ca^2+^ imaging studies [48], [55], [56] that show a decrease in the rise of intracellular Ca^2+^ concentration during an action potential that back-propagates along the apical dendrite [54]. It has been argued that the decrease in the peak Ca^2+^ concentration along the apical dendrite may be due to surface to volume ratio or to a decrease in the density of voltage-gated Ca^2+^ channels as a function of distance from the soma along the apical dendrite. Currently, the spatial distribution of the various voltage-gate Ca^2+^ channels along the apical dendrite is unknown. Once a conductance gradient of these important channels will be established it may be possible to provide a better model for dendritic Ca^2+^ spike initiation. Given the similarity between the results presented in Figure 7 and Ca^2+^ imaging studies [48], [55], [56] it may be possible to speculate that the Ca^2+^ channel sub-types are homogenously distributed over the apical dendrite of the L5 pyramidal neurons and the soma membrane. Thus, the decrease in the Ca^2+^ influx may only be the result of the smaller activation of voltage-gated Ca^2+^ conductance by a progressively smaller back-propagating AP. Clearly, further exploration of the dendrites of the L5 pyramidal neurons to pharmacologically and kinetically determine the distribution of the different Ca^2+^ channel sub-types is required in order to test this currently experimentally un supported speculation.
